# Global prevalence of Duchenne and Becker muscular dystrophy: a systematic review and meta-analysis

**DOI:** 10.1186/s13018-022-02996-8

**Published:** 2022-02-15

**Authors:** Nader Salari, Behnaz Fatahi, Elahe Valipour, Mohsen Kazeminia, Reza Fatahian, Aliakbar Kiaei, Shamarina Shohaimi, Masoud Mohammadi

**Affiliations:** 1 Department of Biostatistics, School of Health, Kermanshah University of Medical Sciences, Kermanshah, Iran; 2 Student Research Committee, Kermanshah University of Medical Sciences, Kermanshah, Iran; 3 Department of Medical Genetics, Faculty of Medicine, Tehran University of Medical Sciences, Tehran, Iran; 4 Department of Neurosurgery, School of Medicine, Kermanshah University of Medical Sciences, Kermanshah, Iran; 5 Department of Computer Engineering, Sharif University of Technology, Tehran, Iran; 6 Department of Biology, Faculty of Science, University Putra Malaysia, Serdang, Selangor Malaysia; 7 Cellular and Molecular Research Center, Gerash University of Medical Sciences, Gerash, Iran

**Keywords:** Muscular dystrophy, Duchenne, Becker, Systematic review, Meta-analysis

## Abstract

**Background:**

A variety of mutations in the largest human gene, dystrophin, cause a spectrum from mild to severe dystrophin-associated muscular dystrophies. Duchenne (DMD) and Becker (BMD) muscular dystrophies are located at the severe end of the spectrum that primarily affects skeletal muscle. Progressive muscle weakness in these purely genetic disorders encourages families with a positive history for genetic counseling to prevent a recurrence, which requires an accurate prevalence of the disorder. Here, we provide a systematic review and meta-analysis to determine the prevalence of DMD and BMD worldwide.

**Method:**

The current systematic review and meta-analysis was carried out using Cochrane seven-step procedure. After determining the research question and inclusion and exclusion criteria, the MagIran, SID, ScienceDirect, WoS, ProQuest, Medline (PubMed), Embase, Cochrane, Scopus, and Google Scholar databases were searched to find relevant studies using defined keywords and all possible keyword combinations using the AND and OR, with no time limit until 2021. The heterogeneity of studies was calculated using the *I*^2^ test, and the publication bias was investigated using the Begg and Mazumdar rank correlation test. Statistical analysis of data was performed using Comprehensive Meta-Analysis software (version 2).

**Results:**

A total of 25 articles involving 901,598,055 people were included. The global prevalence of muscular dystrophy was estimated at 3.6 per 100,000 people (95 CI 2.8–4.5 per 100,000 people), the largest prevalence in the Americans at 5.1 per 100,000 people (95 CI 3.4–7.8 per 100,000 people). According to the subgroup analysis, the prevalence of DMD and BMD was estimated at 4.8 per 100,000 people (95 CI 3.6–6.3 per 100,000 people) and 1.6 per 100,000 people (95 CI 1.1–2.4 per 100,000 people), respectively.

**Conclusion:**

Knowing the precise prevalence of a genetic disorder helps to more accurately predict the likelihood of preventing its occurrence in families. The global prevalence of DMD and BMD was very high, indicating the urgent need for more attention to prenatal screening and genetic counseling for families with a positive history.

## Background

Dystrophin-associated muscular dystrophies include a spectrum of recessive X-linked muscle diseases that result from mutations in the dystrophin gene. Dystrophin is mainly expressed in skeletal and cardiac muscles with a well-known role in protecting muscle fibers [1]. Thus, absent or dysfunctional dystrophin primarily affects the skeletal and cardiac muscles, where dystrophin and dystrophin-associated proteins work together to stabilize muscle fibers during contraction and relaxation [2]. Dystrophin is also expressed in some brain cells and this is an explanation for cognitive impairment in patients [2].

Duchenne muscular dystrophy (DMD) is a severe form of dystrophin-associated muscular dystrophies, with early childhood onset [3, 4]. An affected child is usually diagnosed before the age of 4 years with early signs including delayed ability to sit, difficulty rising and standing independently, and difficulties learning to speak. Rapidly progressive muscle weakness in DMD leads to being wheelchair dependent by age 12 years and death before the third decade. Common causes of death in DMD include respiratory complications and heart failure from progressive cardiomyopathy [5–7].

Becker muscular dystrophy (BMD) is usually characterized by a milder and more varied phenotype. The detrimental effect of mutations in the dystrophin gene leading to disease 1 is less than that of Duchenne dystrophy, in that dystrophin may be produced but in smaller amounts or with reduced function [8, 9]. Skeletal muscle weakness in BMD is slowly progressive, with later onset at around age 8. In patients with less cardiac involvement, onset is around age 15. Heart failure is the most common cause of death in BMD [10].

Dystrophin-associated muscular dystrophies are inherited in an X-linked recessive pattern [11]. Due to the recessive inheritance pattern, these disorders are more common in males, but females with a defective allele may also show mild symptoms. Although the rate of new mutations in the huge dystrophin gene is high, about two-thirds of cases are diagnosed with a carrier mother. DMD or BMD mutations are inherited with a 50% probability in each pregnancy of a carrier mother. Males who inherit the mutation develop the disease, while the onset of symptoms in carrier females depends on the pattern of X-inactivation. Genetic testing on a muscle biopsy can detect DMD with about 95% accuracy [12–14]. Also, electromyography shows muscle degeneration in affected individuals. Affected individuals exhibit extremely high levels of creatine kinase in the bloodstream. Elevated creatine kinase levels are also seen in some carrier females [14].

There is no known treatment for dystrophin-associated muscular dystrophies. These disorders negatively affect the quantity and quality of life of patients and increase the cost of health care for the family and society; therefore, prevention is very important [14, 15]. To predict the likelihood of having an affected child in genetic counseling before pregnancy, it is important to know information such as the probability that the mother is a carrier, frequency of carriers with increased creatine kinase, the possibility of inheriting the defective allele, and the exact prevalence of the disease worldwide [16, 17]. The prevalence of a genetic disease may change due to the growing awareness of people about genetic diseases and the encouragement of carriers of recessive disorders for prenatal screening [15, 18]. Although various studies have been performed to determine the prevalence of DMD and BMD in different populations and countries, there is no comprehensive study reporting the overall prevalence of these disorders worldwide. Therefore, the present study was conducted to determine the prevalence of DMD and BMD worldwide in a systematic review and meta-analysis.

## Methods

The present systematic review and meta-analysis was conducted based on the Cochrane method which includes seven steps: selecting research question, determining inclusion and exclusion criteria, collecting articles, selecting the desired articles, evaluating the quality of articles, data extraction, analysis, and interpretation of findings.

### Determine research question and keywords

The research question was designed as "What is the is the prevalence of DMD and BMD worldwide?". Therefore, the study population included patients with DMD or BMD. Finding; the prevalence of DMD and BMD, time limitation; from the date of publication of the first related article until March 6, 2021, and type of studies; descriptive cross-sectional. Keywords were extracted from the MeSH browser. Keywords related to the studied population (P) included Muscular Dystrophies, Muscular Dystrophy, Duchenne Muscular Dystrophy, Becker Muscular Dystrophy. The keyword related to outcome (O) was prevalence.

### Inclusion and exclusion criteria according to the research question

Since the purpose of this study is to determine the population-based prevalence, cross-sectional studies, which are the most studies reporting population-based prevalence, were selected as the study that meets the inclusion criteria. According to this the study included descriptive cross-sectional studies published in Persian or English, with full text available and reporting the prevalence of DMD or BMD in different parts of the world in genetics context [19]. The unrelated analytical, cohort studies, case–control, and conference studies, report studies, case series, review studies, intervention, and clinical trial studies were excluded.

### Identifying articles

Two Persian databases including MagIran and SID, and five international databases including ScienceDirect, Web of Science (WoS), ProQuest, Medline (PubMed), Embase, Cochrane, and Scopus were searched to find articles related to the research question. The Google Scholar search engine was also used for the final search. To retrieve the relevant articles, no time limit was set and all published articles until March 6, 2021, were reviewed. The search was limited to published Persian and English articles. All possible combinations of keywords were used through AND and OR in the advanced search of all mentioned databases. For example, in the PubMed database, the search strategy was determined as follows: ((((Muscular Dystrophies [Title/Abstract]) OR (Muscular Dystrophy [Title/Abstract])) OR (Duchenne Muscular Dystrophy [Title/Abstract])) OR (Becker Muscular Dystrophy [Title/Abstract])) AND (Prevalence [Title/Abstract]).

Evaluations in this study were performed independently and blinded. Initially, two researchers (MK and BF) reviewed the titles and abstracts of articles (according to inclusion criteria) according to PRISMA 2009 checklist; in case of disagreement among the researchers regarding each of the articles, the third party (MM) reviewed and provided the final opinion regarding that study. An alert was created on a number of important databases including PubMed and Scopus, to access the latest published articles to review new articles published during the study. In addition, all references of articles in line with inclusion criteria were manually reviewed to access all relevant articles.

### Selection of articles based on inclusion and exclusion criteria

The review process of articles in this study was based on the 4-step PRISMA 2009 process based on the sections of article identification, article screening, review of entry, and exit criteria for studies and articles submitted to the meta-analysis in the final stage.

Article information was transferred to the Endnote X8 software. Duplicate articles were removed after searching all databases. The names of the authors and the titles of the journals were then removed to reduce the possibility of bias in article selection. A checklist was created based on the title and abstract of the articles. Then, two researchers (MK. and BF) reviewed the title and abstract of the articles separately and excluded nonresearch studies, unrelated to the inclusion and exclusion criteria, and also studies without access to their full text. Then, the full text of the remaining articles was reviewed to exclude studies that did not meet the inclusion criteria.

### Qualitative assessment of articles

Qualitative assessment was performed using the STROBE checklist which is a useful method for qualitative assessment of descriptive research. Articles are scored according to this checklist, including minimum and maximum scores of 0 and 32, respectively. Articles with a score of ≥ 16 were considered as a medium- to high-quality studies and were included in the systematic review and meta-analysis, while articles with a score below 16 were excluded [20].

### Data extraction

Then, a summary of selected studies was prepared, including the surname of the first author, year of publication, place of study, sample size, age of subjects, type of MD, and its prevalence per 100,000 people.

### Statistical Analysis

The prevalence of DMD and BMD in each study was considered as a probability of binomial distribution, and its variance was measured by binomial distribution to analyze and combine the results of different studies. The heterogeneity of studies was assessed using the *I*^2^ test. The random-effects model was used when the *I*^2^ index was greater than 50%. Since parameter variations between studies are considered in the calculations in this model, it can be said that the findings in heterogeneous conditions are more generalizable than the model with a fixed impact. The publication bias was assessed using the Begg and Mazumdar rank correlation tests. The significance level of the test was P (0.1). Data analysis was performed using comprehensive Meta-Analysis (Version 2) software. Sensitivity analysis was performed to evaluate the impact of each study on the final result.

## Results

**Fig. 1:**
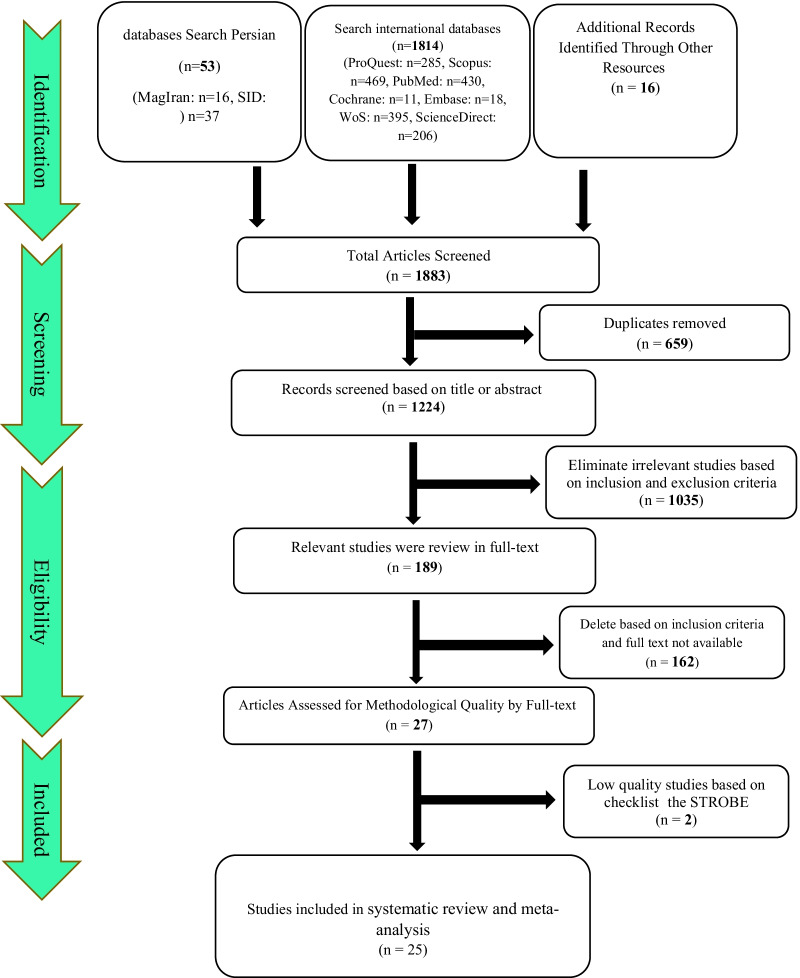
Flowchart indicating the stages of article selection in this systematic review and meta-analysis (PRISMA 2009)

Initially, 1883 articles were retrieved, including 1814 articles from international databases, 53 articles from Persian databases, and 16 articles from references. In total, 1883 articles were excluded, of which 659 articles due to being duplicated, 1035 articles with title and abstract review, and 162 articles with the full-text review. Qualitative evaluation of the remaining 27 articles was performed using the STROBE checklist, two of which had poor methodological quality and were excluded. Finally, 25 articles entered the process of systematic review and meta-analysis (Fig. 1).

### General characteristics of the final included articles

The total sample size of all articles was 90,159,805 people. The final included articles were published between 1982 and March 6, 2021. The study by El-Tallawy et al. (2005) was conducted with the lowest sample size of 52,203 people in Egypt [44]. The study by Ballo et al. (1994) with a maximum sample size of 15,092,000 people was conducted in South Africa [43]. Table 1 shows a summary of the characteristics of the included articles (Table 1).

**Table 1:** Summary of study specifications

First author, year, references	Type of muscular dystrophy	Prevalence per hundred thousand people	Sample size	Diagnostic criteria	Age (years)	Country	Report year
Nakagawa-1,1991, [21]	DMD	7.12	603,392	Clinical presentation, high serum CK levels, EMG,	-	Japan	1989
Nakagawa-2,1991, [21]	BMD	1.82	603,392	Clinical presentation, high serum CK levels, EMG,	-	Japan	1989
Chan,2015, [22]	DMD/BMD	10.3	873,786	–	0–24	China	2015
Chung-1,2003, [23]	DMD	10.44	631,854	High serum CK level, nerve conduction study, EMG, muscle biopsy, genetic testing	–	China	2001
Chung-2,2003, [23]	BMD	1.26	631,854	–	–	China	2001
Talkop,2003, [24]	DMD	12.76	195,869	–	> 20	Estonia	1998
Lefter-1,2017, [25]	DMD	3	1,666,666	Genetic and electrophysiological tests	–	Ireland	2017
Lefter-2,2017, [25]	BMD	2.2	1,681,818	Genetic and electrophysiological tests	–	Ireland	2017
Husebye-1,2020, [26]	DMD	2.01	547,263		–	Norway	2020
Husebye-2,2020, [26]	BMD	0.04	500,000		–	Norway	2020
Siciliano-1,1999, [27]	DMD	1.69	1,296,275	Genetic testing, clinical examination, high serum CK levels, family history, muscle biopsy	13.8 ± 6.7	Italy	1997
Siciliano-2,1999, [27]	BMD	2.46	1,296,275	Genetic testing, clinical examination, high serum CK levels, family history, muscle biopsy	36.3 ± 16.5	Italy	1997
Peterlin-1,1997, [28]	DMD	2.9	1,034,482	Clinical picture, serum enzymes, EMG and muscle biopsy	–	Slovenia	1997
Peterlin-2,1997, [28]	BMD	1.2	1,000,000	Clinical picture, serum enzymes, EMG and muscle biopsy	–	Slovenia	1997
Mostacciuolo-1,1993, [29]	DMD	3.31	2,296,072	–	–	Italy	1993
Mostacciuolo-2,1993, [29]	BMD	2.01	1,044,776	–	–	Italy	1993
Bushby,1991, [30]	BMD	2.37	3,070,000	–	11	UK	1988
Hughes-1,1996, [31]	DMD	4.25	1,573,282	–	–	Ireland	1994
Hughes-2,1996, [31]	BMD	1.58	1,573,282	–	–	Ireland	1994
Jeppesen,2003, [32]	DMD	5.49	2,636,364	–	–	Denmark	1985–2002
Norwood-1,2009, [33]	DMD	8.29	1,495,778	Genetic testing and genetic investigations	–	England	2007
Norwood-2,2009, [33]	BMD	7.28	1,495,778	Genetic testing and genetic investigations	–	England	2007
Darin-1,2000, [34]	DMD	16.7	185,004	Clinical examinations, high serum CK levels, family history, muscle biopsy, genetic testing	–	Sweden	1995
Darin-2,2000, [34]	BMD	1.62	185,004	Clinical examinations, high serum CK levels, family history, muscle biopsy, genetic testing	–	Sweden	1995
Danieli,1977, [35]	DMD	3.4	3,000,000	High serum CK levels	–	Italy	1952–1972
Ahlström,1977, [36]	DMD	0.7	285,714	–	11	Sweden	1988
Rasmussen-1,2012, [37]	DMD	16.2	1,654,670	Genetic testing and/or muscular biopsy	–	Norway	2005
Rasmussen-2,2012, [37]	BMD	3.5	1,654,670	Genetic testing and/or muscular biopsy	–	Norway	2005
van Essen,1992, [38]	DMD	5.4	7,102,598	Clinical status, serum CK levels, EMG, muscle biopsy	–	Netherlands	1961–1982
LETH,1985, [39]	DMD	6.94	2,348,703	Histological changes in muscular tissue, typical electromyographic changes, high serum CK levels	–	Denmark	1965–1975
MONCKTON-1,1982, [40]	DMD	3.12	705,128	–	–	Canada	1962
MONCKTON-2,1982, [40]	DMD	9.5	989,473	–	–	Canada	1979
Romitti-1,2015, [41]	BMD	3.6	3,827,532	ICD-9 CM code: 359.1 or ICD-10CM code: G71.0	5–24	USA	1982–2011
Romitti-2,2015, [41]	DMD	10.16	3,827,532	ICD-9 CM code: 359.1 or ICD-10CM code: G71.0	5–24	USA	1982–2011
Mah-1,2011, [1]	DMD	10.6	4,990,566	Clinical phenotypes, diagnostic methods, molecular genetic reports	–	Canada	2000–2009
Mah-2,2011, [1]	BMD	2.74	4,990,566	Clinical phenotypes, diagnostic methods, molecular genetic reports	–	Canada	2000–2009
Ramos-1,2016, [42]	DMD	5.17	1,757,189	–	5.5	Puerto Rico	2012
Ramos-2,2016, [42]	BMD	2.84	1,757,189	–	9	Puerto Rico	2012
Ballo-1,1994, [43]	DMD	0.9	15,092,000	High serum CK levels, EMG, genetic testing	–	South Africa	1987–1992
Ballo-2,1994, [43]	BMD	0.13	15,092,000	High serum CK levels, EMG, genetic testing	–	South Africa	1987–1992
El-Tallawy-1,2005, [44]	DMD	7.66	52,203	High serum CK levels investigations, genetic testing, muscle biopsy	–	Egypt	1997
El-Tallawy-2,2005, [44]	BMD	3.83	52,203	High serum CK levels investigations, genetic testing, muscle biopsy	–	Egypt	1997
Radhakrishnan,1987, [45]	DMD	5.99	516,667	Clinical examination, family history, serum CPK, EMG	8.2 ± 3	Libya	1987

**Fig. 2:**
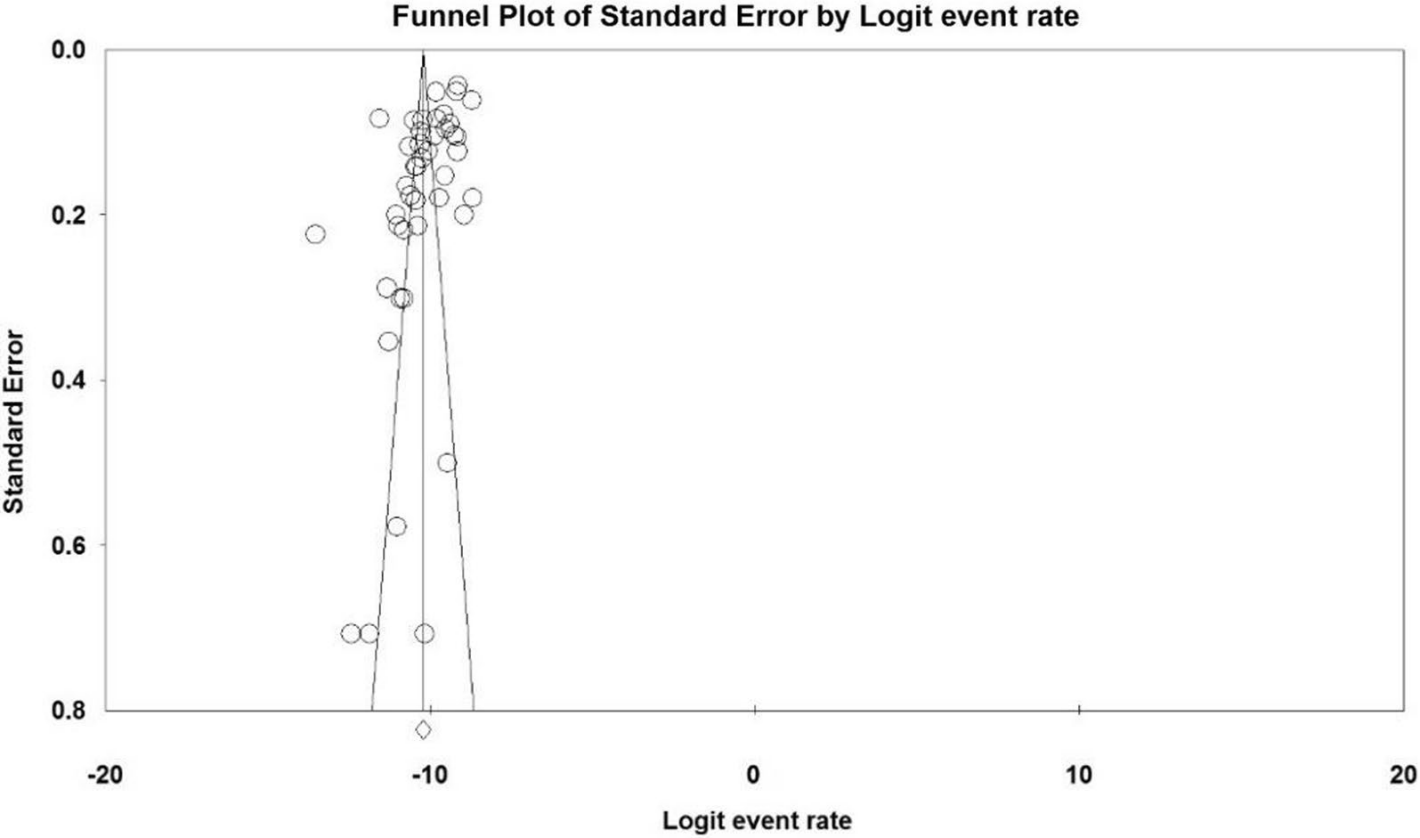
Results of the funnel plot to estimate the prevalence of MD worldwide

*I*^2^ test for the prevalence of MD in different parts of the world showed that there is significant heterogeneity between studies (*I*^2^ = 97.9). Therefore, the data were analyzed using meta-analysis and a stochastic effects model. Sensitivity analysis was also performed to evaluate the effect of each study on the final result and the degree of heterogeneity. According to the Begg and Mazumdar rank correlation test with a significance level of less than 0.1 (*P* = 0.209), there was no publication bias in the inclusion of studies (Fig. 2).

**Fig. 3:**
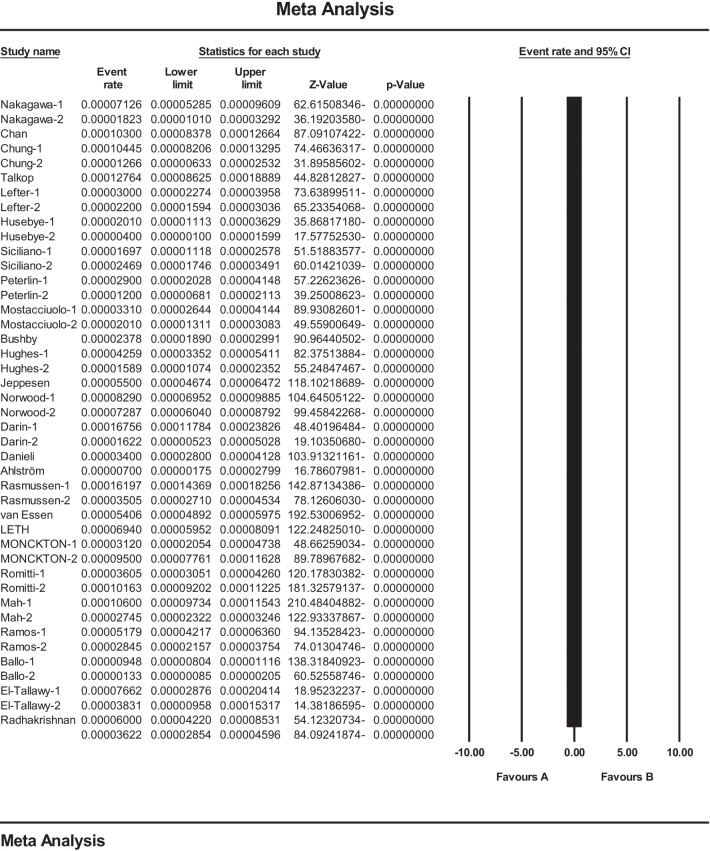
Results of the forest plot to estimation of the prevalence of MD worldwide based on a random-effects model

Combining studies based on a random-effects model, the overall estimation of the prevalence of MD in the world was 3.6 per 100,000 people (95 CI 2.8–4.5). In each study, the black square represents the prevalence, and the length of the line segment on the square indicates a 95 CI. For all studies, the diamond shows a worldwide prevalence (Fig. 3).

**Fig. 4:**
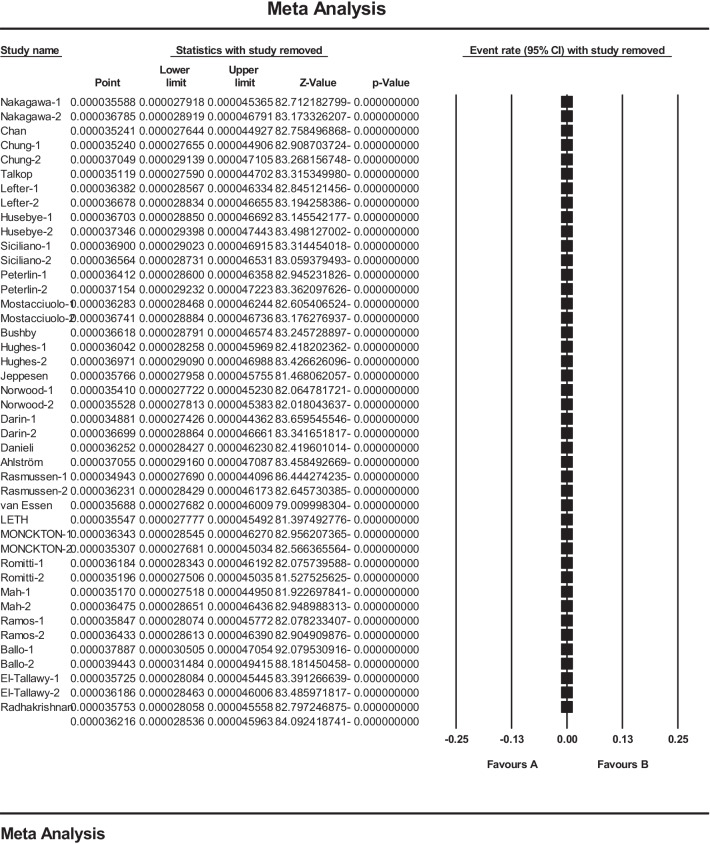
Results of sensitivity analysis in studies reviewed in meta-analysis

In this study, in order to ensure stable results and stability of the results presented in the meta-analysis, sensitivity analysis was used and the results are reported in Fig. 4 and show that after deleting the results of each study, there is no significant change in the final meta-analysis (Fig. 4).

Table 2 shows the analysis of continental subgroups (Asia, Europe, Africa, and Americas) based on various reports of the prevalence of MD in different parts of the world. The Americas showed the highest prevalence of MD with 5.1 per 100,000 people (95 CI 3.4–7.8) (Table 2). In addition, the estimated overall prevalence of DMD and BMD worldwide was 4.8 per 100,000 people (95 CI 3.6–6.3) and 1.6 per 100,000 people (95 CI 1.1–2.4) with a significant publication bias in BMD results, respectively (Table 3).

**Table 2:** Analysis of continental subgroups to estimate the prevalence of MD

Continent	*N*	Sample size	*I*^2^	Prevalence (95% CI)
Asia	5	3,344,278	93.5	4.8 (95% CI 2.7–8.6)
Europe	25	40,820,343	96.8	3.5 (95% CI 2.7–4.7)
America	8	15,190,111	98.2	5.1 (95% CI 3.4–7.8)
Africa	5	30,805,073	98	1.7 (95% CI 1.1–4.5)

**Table 3:** Analysis of continental subgroups to estimate the prevalence of DMD and BMD

Type	*N*	Sample size	*I*^2^	Prevalence (95% CI)
DMD	25	52,657,212	97.7	4.8 (95% CI 3.6–6.3)
BMD	17	36,628,807	95.5	1.6 (95% CI 1.1–2.4)

## Discussion

Muscular dystrophies are inherited, heterogeneous group of disorders caused by mutations in a number of genes that encode proteins involved in supporting muscle cell stability. Loss of function or dysfunction of these proteins leads to gradual weakness and degeneration of muscles, especially skeletal and cardiac muscle [46, 47]. There is a variety of MD that DMD and BMD are the most common forms after myotonia [46]. The age of onset and severity of symptoms in DMD and BMD are different, which is explained by the different effects that different mutations have on the dystrophin protein. Rapidly progressing muscle degeneration in DMD patients causes symptoms around the age of 3 and loss of ability to stand and move and wheelchair dependence around the age of 13 years [8]. Another threatening issue is the involvement of the cardiorespiratory system, which is the leading cause of death in these patients. Some patients also suffer from behavioral and cognitive disorders, mental disability, attention-deficit/hyperactivity disorder (ADHD), and autism spectrum disorders [48].

The impact of these disorders on the lifespan and quality of life suggests the need for preventive measures for the birth of an affected child. Due to the recessive, X-dependent nature, the sons of a carrier mother inherit defective allele with a 50% chance and become affected. Although carrier girls are usually asymptomatic or show only high levels of creatine kinase, some cases develop symptoms depending on the X-inactivation pattern. One of the important data to estimate more accurately the transmission risk of a recessive allele is the prevalence of disorder worldwide. Parental awareness, active genetic counseling centers, carrier screening, prenatal screening, the growing propensity for unrelated marriages, and the tendency to have fewer children in some populations influence the prevalence of recessive genetic disorders.

In the present systematic review and meta-analysis, the overall prevalence of MD, DMD, and BMD worldwide was estimated at 3.6, 4.6, and 1.6 per 100,000 people, respectively. The highest and lowest prevalence of DMD was reported in the study of Darin et al. with 16.7٪ [34] and the study of Ahlström et al. with 0.7٪ [36], respectively. Also, the highest and lowest prevalence of BMD was reported in the study of Norwood et al. [33] with 7.28 per 100,000 people and Husebye et al. [26] with 0.04 per 100,000 people, respectively.

In the systematic review and meta-analysis of Mah et al. (2014), the prevalence of DMD and BMD was 4.78 and 1.53 per thousand, respectively [13]. Also, Theadom et al. (2014) reported the prevalence of DMD and BMD of 1.7–4.2 and 0.4–3.6 per thousand, respectively [14]. Our results were almost similar to these studies, and minor differences could be due to the inclusion of more articles and patients from different races and geographies around the world.

According to the various reports of the prevalence of DMD and BMD in different countries as well as changes in the population structure in different parts of the world, a nationwide analysis is needed in different countries for the prevalence of the disorders. Also, the relevant authorities need to pay more attention to the prevalence of the disorders, preventive strategies, and treatment strategies to increase the lifespan and improve the quality of life of patients. As a result of the subgroup study, the Americas have the highest prevalence of 5.1 per 100,000 people (95 CI 3.4–7.8 per 100,000 people), while Africa has the lowest prevalence of 1.7 percent per 100,000 people (95 CI 1.1–4.5 per 100,000 people).

The study by Ballo et al. (1994) in South Africa [43] had the highest sample size and reported a prevalence of DMD of 0.9 per 100,000 and BMD of 0.13 per 100,000, which differs from the overall results of the present study. The funnel plot was also used to demonstrate publication bias in studies included in the meta-analysis process.

DMD and BMD are highly prevalent worldwide and have many negative consequences for the individual and society, including the economic costs that increase significantly as the diseases progress [49]. Therefore, identifying patients and proper management to improve the quality of life of patients and their families, and help achieve better therapies, the use of supportive therapies to reduce the symptoms of the disease seems useful. A better understanding of the prevalence and age of onset of DMD and BMD is crucial because this knowledge can provide valuable information to healthcare providers that enriches healthcare interventions and enhances service quality [50, 51].

There are a variety of muscular dystrophies that are different in symptoms, severity, age of onset, and prevalence. Proteins involved in various forms of muscular dystrophy usually work together and all work to maintain the stability of muscle fibers. Various mutations in the genes encoding these proteins can lead to different phenotypes or different phenotypic severity, which sometimes makes it difficult to diagnose the exact type of disease. Also, genetic variations between populations or ethnic groups can alter the reported prevalence of muscular dystrophy. The development of existing molecular diagnostic tools as well as new diagnostic strategies leads to more accurate diagnosis of cases. Furthermore, the availability of information such as hospital charts and other sources of medical information to determine the number of diagnosed cases of relatively uncommon diseases such as muscular dystrophies varies among countries.

Moreover, different approaches of case ascertainment have been used in different studies and methodological uniformity is not mainly seen. Also, a number of studies have reported an estimated prevalence of the disease in a particular population in a language other than English, for example in the present study only Persian and English articles were included. Therefore, significant gaps remain in estimating the global prevalence of many types of muscular dystrophy. Apart from these, this systematic review and meta-analysis faced limitations such as unavailability of the full text of some articles, variability in methodology, lack of genetic testing in earlier studies that may affect accurate estimation of the disease prevalence, and nonrandom geographic distribution. Overall, additional epidemiological studies are suggested to more accurately estimate the prevalence of muscular dystrophies, particularly DMD and BMD worldwide, using standardized diagnostic criteria and multiple case ascertainment approaches will help to estimate the economic impact and burden of patient health care.

## Data Availability

Datasets are available through the corresponding author upon reasonable request.

## Abbreviations

SID: Scientific Information Database
MESH: Medical Subject Headings
WoS: Web of Science
PRISMA: Preferred Reporting Items for Systematic Reviews and Meta-Analysis
STROBE: Strengthening the reporting of observational studies in epidemiology for cross-sectional study
DMD: Duchenne muscular dystrophy
BMD: Becker muscular dystrophy
